# Neoadjuvant chemotherapy with gemcitabine plus cisplatin followed by radical liver resection versus immediate radical liver resection alone with or without adjuvant chemotherapy in incidentally detected gallbladder carcinoma after simple cholecystectomy or in front of radical resection of BTC (ICC/ECC) – a phase III study of the German registry of incidental gallbladder carcinoma platform (GR)– the AIO/ CALGP/ ACO- GAIN-trial –

**DOI:** 10.1186/s12885-020-6610-4

**Published:** 2020-02-14

**Authors:** Thorsten O. Goetze, Wolf O. Bechstein, Ulli Simone Bankstahl, Tobias Keck, Alfred Königsrainer, Sven A. Lang, Claudia Pauligk, Pompiliu Piso, Arndt Vogel, Salah-Eddin Al-Batran

**Affiliations:** 1 Institut für Klinisch-Onkologische Forschung (IKF), Krankenhaus Nordwest gGmbH, Frankfurt am Main, Germany; 2 Klinik für Allgemein- und Viszeralchirurgie, Universitätsklinikum Frankfurt, Frankfurt am Main, Germany; 3 Klinik für Chirurgie, Universitätsklinikum Schleswig-Holstein, Lübeck, Germany; 4 Klinik für Allgemeine, Viszeral- und Transplantationschirurgie, Universitätsklinik Tübingen, Tübingen, Germany; 5 Klinik für Allgemein- und Viszeralchirurgie, Universitätsklinikum Freiburg, Freiburg im Breisgau, Germany; 6 Klinik für Allgemein- und Viszeralchirurgie, Barmherzige Brüder Regensburg, Regensburg, Germany; 7 Klinik für Gastroenterologie, Hepatologie und Endokrinologie, Medizinische Hochschule Hannover, Hannover, Germany

**Keywords:** Gallbladder carcinoma, Biliary tract cancer, Neoadjuvant therapy, Perioperative chemotherapy, Randomized trial

## Abstract

**Background:**

Currently, complete surgical resection represents the only potentially curative treatment option for Biliary Tract Cancer (BTC) including Gallbladder Cancer (GBC). Even after curative resection, 5-year OS is only 20–40%. Gallbladder carcinoma is relatively rare, but still the fifth most common neoplasm of the digestive tract and even the most frequent cancer of the biliary system. Gallbladder carcinoma is suspected preoperatively in only 30% of all pts., while the majority of cases are discovered incidentally by the pathologist after cholecystectomy for a benign indication. For improving curative rates in BTC and GBC, early systemic therapy combined with radical resection seems to be a promising approach. The earliest moment to apply chemotherapy would be in front of radical surgery. The encouraging results of neoadjuvant/perioperative concepts in other malignancies provide an additional rationale to use this treatment in the early phase of GBC management and even ICC/ECC. Especially because data regarding pure adjuvant chemotherapy in BTC’s are conflicting.

**Methods:**

This is a multicenter, randomized, controlled, open-label phase III study including pts. with incidentally discovered GBCs after simple cholecystectomy in front of radical liver resection and pts. with resectable/ borderline resectable cholangiocarcinomas (ICC/ ECC) scheduled to receive perioperative chemotherapy (Gemcitabine + Cisplatin 3 cycles pre- and post-surgery) or surgery alone followed by a therapy of investigator’s choice. Primary endpoint is OS; secondary endpoints are PFS, R0-resection rate, toxicity, perioperative morbidity, mortality and QoL. A total of *N* = 333 patients with GBC or BTC will be included. Recruitment has started in August 2019.

**Discussion:**

The current proposed phase III GAIN study investigates whether induction chemotherapy followed by radical resection in ICC/ECC and re-resection in IGBC (and – if possible – postoperative chemotherapy) prolongs overall survival compared to radical surgery alone for incidental gallbladder carcinoma and primary resectable or borderline resectable cholangiocarcinoma. Utilizing a neoadjuvant approach including a second radical surgery will help to raise awareness for the necessity of radical surgery, especially second radical completion surgery in IGBC and improve the adherence to the guidelines.

**Trial registration:**

ClinicalTrials.gov ID: NCT03673072 from 17.09.2018. EudraCT number: 2017–004444-38 from 02.11.2017.

## Background

Biliary tract cancer is a rare malignancy arising from epithelial cells of the biliary tree. Cholangiocarcinoma (CCA) is associated with poor prognosis and standard therapeutic options are limited. The global incidence varies according to geographical region with a significantly higher burden in South-East Asia compared to the western world [1]. Here, the rate of intrahepatic cholangiocarcinoma (ICC) is low with 0.4 to 1.0 cases per 100,000. The highest incidence is observed in patients older than 65 years of age. Incidence and mortality rates are increasing within the last decades in developed countries. In contrast, hilar and distal CCA demonstrate only minor regional variations with incidence rates between 0.5 and 1.1 per 100,000. A minimal male predominance is found in biliary tract cancer patients. Cirrhosis of the liver, infection with hepatitis B and C and primary sclerosing cholangitis are known risk factors [2–5]. The incidence of gallbladder carcinoma (GBCA) is around 2.0 per 100,000 with a median age of 67 years at the time of diagnosis. Gallstones and chronic infections of the gallbladder are the most important risk factors for developing GBCA [6–9].

Gallbladder carcinoma is relatively rare, but still the fifth most common neoplasm of the digestive tract and even the most frequent cancer of the biliary system [10]. Gallbladder carcinoma is suspected preoperatively in only 30% of all patients [11, 12], while the majority of cases are discovered incidentally by the pathologist (IGBC) after cholecystectomy for a benign indication. Reported cases of IGBC in Germany are registered in the “German Registry of Incidental Gallbladder Carcinoma” (GR), the largest casebook of gallbladder carcinomas in Europe [11–21]. The GR shows that surgical treatment of gallbladder carcinoma patients remains not adequate despite widely published guidelines [13]. Less than 50% of the patients received stage adjusted surgical therapy according to the GR data [22]. Stage adjusted therapy according to the NCCN-, ESMO- and German S3- Guidelines contains liver resection combined with dissection of the regional lymph nodes along the hepatoduodenal ligament in cases of T1b, or more advanced carcinomas [23, 24]. Gallbladder neoplasms shows a high incidence of locoregional failure after surgical resection, with early spread to celiac, retropancreatic, and aortocaval nodes as well occult liver spread [25] in formally R0 patients after simple cholecystectomy (SC). The rate of positive lymphatic nodes is 31.2% in T2- and 45.5% in T3-stage carcinomas [25, 26]. Lymphatic spread beyond the hepatoduodenal ligament generally represents distant metastatic disease, and a cure of such patients by a pure surgical concept does not seem to be achievable.

Therefore, there is a need for a systemic therapy as early as possible in the course of treatment in BTC (ICC/ECC) and also in IGBC’s.

The landmark trial, UK ABC-02 by Valle et al. [27] compared gemcitabine/cisplatin with gemcitabine alone in locally advanced or metastatic cholangio- and gallbladder carcinomas and showed clear superiority of the combination, with significant improvements for PFS (8 vs. 5 months, *p* < 0.001) and OS (8.1 vs. 11.7 months, *P* < 0.001). Basically, the study indicates the sensitivity of this disease towards chemotherapy and provides a rationale for the use of this chemotherapeutic doublet in the present study.

For improving disease control and cure rates in BTC (ICC/ ECC) and of IRR in T2–3 IGBC’s, it is meaningful to implement early additional systemic therapy. The earliest moment to apply chemotherapy would be directly after simple cholecystectomy in IGBC’s and right before surgery in ICC/ECC. The encouraging results of neoadjuvant/perioperative concepts in esophagogastric, stomach, rectal, and other malignancies provide an additional rationale to use this treatment in the early phase of IGBC management and even ICC/ECC. However, due to the fact that 2/3 of gallbladder carcinomas are incidental findings after simple cholecystectomy, an earlier start of a systemic therapy in IGBC will be not realizable. Furthermore, preoperatively discovered gallbladder carcinomas are usually too advanced for neoadjuvant/perioperative concepts.

## Methods/design

### Protocol overview

GAIN is a multicenter, randomized, controlled, open-label phase III study including patients with pT2–3 N- or pT1-3 N+ incidentally discovered gallbladder carcinomas (IGBC/ 70% of all GBC’s) after simple cholecystectomy and patients with resectable/ borderline resectable cholangiocarcinomas (ICC/ ECC) scheduled to receive perioperative chemotherapy or surgery alone. Study sponsor following German Pharmaceuticals Act is the Krankenhaus Nordwest gGmbH (Frankfurt), lead coordinating investigator is PD Dr. Thorsten O. Goetze.

Potential study participants will be assessed for eligibility during a 28-day screening period. Eligible patients will be enrolled and randomized to perioperative chemotherapy (Arm A) or immediate surgery alone with or without adjuvant chemotherapy (investigator’s choice) (Arm B). Randomization will occur in a 1:1 ratio with stratification by clinical tumor stage (T1 and T2 vs. T3 and T4), ECOG (0 and 1 vs. 2) and localization of the primary (ICC vs. ECC vs. IGBC).

Neoadjuvant chemotherapy with gemcitabine plus cisplatin will be administered for 3 cycles preoperatively followed by radical liver resection versus immediate radical liver resection alone with or without adjuvant chemotherapy (investigator’s choice) in incidentally detected T2–3N- or pT1-3 N+ gallbladder carcinoma after simple cholecystectomy or in front of radical resection of Biliary Tract Cancer (ICC/ECC). After the radical tumor resection again 3 cycles postoperative chemotherapy will be administered in the experimental arm. In the standard (control) arm no perioperative chemotherapy will be administered. After surgery adjuvant chemotherapy can be administered by investigator’s choice.

#### Arm A

Patients will receive gemcitabine (1000 mg/m^2^) plus cisplatin (25 mg/m^2^) every 3 weeks on days 1 and 8 intravenously. Treatment with gemcitabine plus cisplatin will be administered for 3 cycles preoperatively and for 3 cycles postoperatively. In case of progressive or recurrent disease, unacceptable toxicity, or withdrawal of consent, treatment will be terminated.

#### Arm B

**Fig. 1:**
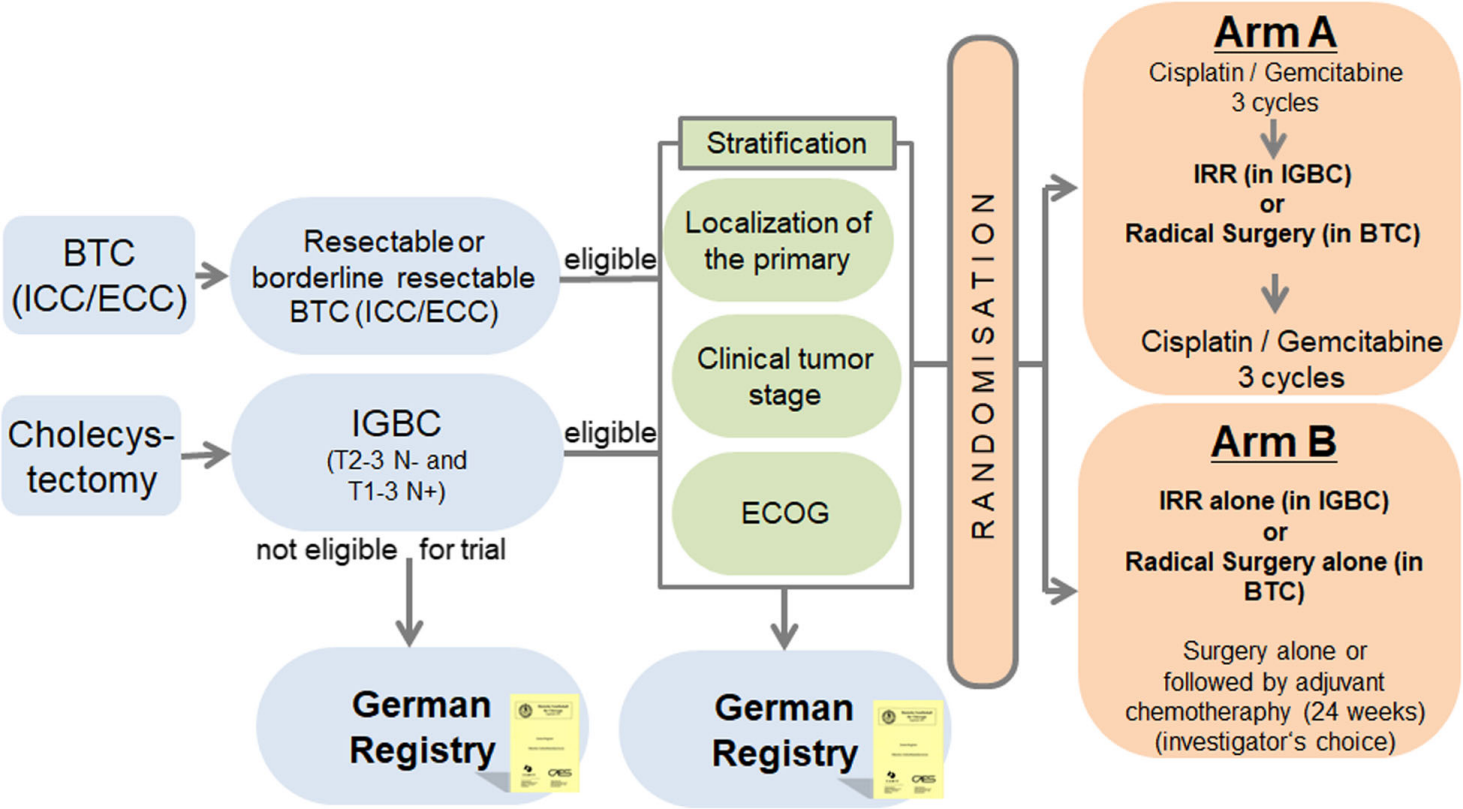
Study Scheme. BTC (ICC/ ECC) = Biliary Tract cancer (Intrahepatic Cholangiocarcinoma/ Extrahepatic Cholangiocarcinoma); IGBC = Incidental Gallbladder Carcinoma; IRR = Immediate Radical Re-resection

Patients will receive surgery immediately, without receiving preoperative chemotherapy. After surgery adjuvant chemotherapy can be administered by investigator’s choice. In both of the treatment arms, tumor assessments (CT or MRI) are performed before randomization and prior to surgery. Therefore, in patients randomized to Arm A (surgery + chemotherapy) there will be an additional staging before the surgical procedure, after completing 3 cycles of chemotherapy. After surgery, tumor assessments are performed every 3 months until progression/relapse, death or end of follow-up. During treatment, clinical visits (blood cell counts, detection of toxicity) occur prior to every treatment dose. Safety of Cisplatin/ Gemcitabine will be monitored continuously by careful monitoring of all adverse events (AEs) and serious adverse events (SAEs) reported. Please see Fig. 1 for reference.

### Measures of outcomes and assessments

#### Primary outcome

The primary endpoint is overall survival. The duration of OS will be determined by measuring the interval from randomization to the date of death or last observation (censored).

#### Secondary outcomes

The main secondary endpoint is QoL. The QoL data will help us to better integrate a possible gain in OS into the therapy guidelines. Other secondary outcome measures are 3-year survival rates as well as the projected 5-year overall survival rate in addition to progression-free survival, toxicity, 30 days and 90 days (perioperative) morbidity and mortality.

#### Main inclusion criteria

Histologically confirmed incidental gallbladder carcinoma (IGBC) (T2–3N- or T1-3N+ after Cholecystectomy) or Biliary tract cancer (BTC) (intrahepatic, hilar or distal Cholangiocarcinoma (CCA)) scheduled for complete resection (mixed tumor entities with hepatocellular carcinoma are excluded). Medical and technical operability of the primary. No prior chemo- therapy and no prior tumor resection, for IGBC (T2–3N- or pT1-3 N+) prior Cholecystectomy is allowed.

#### Main exclusion criteria

Medical inoperability. Exclusion of distant metastases by CT or MRI. Exclusion of the infiltration of any adjacent organs or structures by CT or MRI, indicating an unresectable situation.

### Treatments

#### Control(s)/comparator(s)

Gemcitabine/Cisplatin consists of: Gemcitabine will be administered at a dose of 1000 mg/m^2^ as 0.5 h infusion on D1 and D8 Q3W. Cisplatin will be administered at a dose of 25 mg/m^2^ as 1 h infusion on D1 and D8 Q3W [27].

#### Dose, mode and scheme of intervention

In the interventional arm surgery is planned to occur 4 to 6 weeks after D8 of last gemcitabine plus cisplatin dose (for Arm A) or directly after randomization for Arm B.

The protocol specifications on surgical treatment reflect national guidelines and current expert opinion. Aim of surgical resection is a margin-free (R0) resection of the primary tumor. Hepatic resection should be performed to obtain clear margins. In IGBC a radical re- resection usually consists of wedge resection of segments IVb and V or bisegmentectomy of segments IVb and V as the minimal volume required. Liver resections should always combined with a standardized lymphadenectomy for therapeutic and staging reasons.

#### Sample size calculation

Sample size calculation is based on the results obtained from the German Registry of Incidental Gallbladder Carcinoma Platform (GR) and additional theoretical assumptions to eliminate selection bias. Taken together, the T2 and T3 data, the median OS of the control arm was calculated at 24 months. An improvement of OS according to a HR of 0.70 is clinically relevant and would justify the implementation of a burdensome chemotherapy before major surgery, and is still realistic and within the frame of improvement achieved in other gastrointestinal malignancies by neoadjuvant/adjuvant concepts. Assuming a 24 months median OS, the study will enroll 300 patients (1:1) providing 80% power to detect an improvement in Hazard ratio of 0.70 in terms of OS (as assessed by KM-curves) favoring the experimental arm (log rank test, 1-sided alpha = 0.05). The sample size of *N* = 300 includes an exponentially distributed dropout rate of 10% during the first 3 years of follow-up, resulting in *n* = 272 evaluable patients. The recruitment period is set at 4 years and the total follow up period (calculated from last patient in) of 2 years. All patients will be followed up until end of study, at least 2 years. A total of *n* = 333 patients is planned to be screened for the study with 10% screening failure expected resulting in *n* = 300 randomized patients.

#### Monitoring

All adverse events and severe adverse events occurring after informed consent are recorded in the patient’s electronic case report form by the responsible site staff. Adverse events will be assessed according to the Common Terminology Criteria for Adverse Events (CTCAE) version 5.0. With this data the safety will be monitored continuously by careful monitoring of all adverse events and serious adverse events reported. A compilation of all serious adverse events is sent to lead Ethic, regulatory body and the independent Data Monitoring Committee (IDMC). The IDMC furthermore provides the sponsor with recommendations regarding study modification, continuation or termination. In this process the IDMC may give advice for continuation, changes to the study protocol or termination of the study. The IDMC may claim unplanned interim analyses of any variable and – beyond the aforementioned items – it may ask for any additional activity within the trial if the activity is on behalf of patients’ security.

Premature termination of the study may also be decided if unexpected severe surgical complications occur, more effective therapies become available or if patient enrollment is insufficient. Final decision is made by sponsor representative and the lead coordinating investigator.

It is understood that an outside monitor and other authorized personnel may contact and visit the investigator, and that they will be allowed direct access to source data/documents for trial-related monitoring, audits, IRB review, and regulatory inspection. Direct access is defined as permission to examine, analyze, verify, and reproduce any records and reports that are important to evaluation of a clinical trial. All reasonable precautions within the constraints of the applicable regulatory requirements to maintain the confidentiality of patients’ identities and sponsor’s proprietary information will be exercised. In case of an audit by the sponsor/sponsor representative or an appropriate authority, the investigator will make all relevant documents available.

#### Ethical considerations, information giving and written informed consent

The responsible lead ethics committee approved the protocol of the study on the 11th January 2019 under the identification number FF108/2018. The trial has been registered on the ClinicalTrial.gov website under the identification number NCT03673072 https://clinicaltrials.gov/ct2/show/NCT03673072. The GAIN study complies with the Declaration of Helsinki rules, the principles of Good Clinical Practice guidelines and the Data Protection Act. The trial will also be carried out in compliance to local legal and regulatory requirements. For each patient to be enrolled into the study, an obtaining written informed consent prior to inclusion into the trial is essential.

## Discussion

Currently, complete surgical resection represents the only potentially curative treatment option for Biliary tract cancer (ICC- Intrahepatic- and ECC – Extrahepatic Cholangiocarcinoma) including Gallbladder Carcinoma, and is therefore the treatment of choice if the respective tumor is deemed resectable [8]. However, more than 50% of patients already exhibit unresectable disease at the time of diagnosis [9, 28]. Even after curative resection, 5-year overall survival (OS) is only 20–40% [29–36].

Incidental gallbladder carcinoma (IGBC) is identified after laparoscopic or open cholecystectomy [11–13]. In this case, a second, stage-adjusted surgery for radical cholecystectomy (immediate radical re-resection = IRR), including liver resection and lymph node dissection represents the state of the art, and national guidelines recommend this approach [23, 37]. Nevertheless, there are major problems related to the management of patients with IGBC: First, surgical therapy remains inadequate [16, 38–40], since less than 50% of patients affected in Germany receive the required radical surgery [22]. Second, even after radical surgery, the outcome of patients with sub-serosal or liver invading pT2–3 stage (the majority of the tumors) remains poor [13]. According to the German Registry of Incidental Gallbladder Carcinoma (GR), the 5-year OS of T2 stage is 38% with IRR and 22% without IRR [17, 21]. In T3 carcinomas, the 5-year OS is 18% with IRR and 12% without IRR [17, 21].

5-year survival for completely resected bile duct and GBC patients ranges between 20 and 50%. Locoregional failure is observed in more than 50% of patients, even in absence of residual disease (R0) and provides the justification for the study of adjuvant therapy. Previously, the role of adjuvant systemic chemotherapy for resected biliary tract carcinomas is not clearly defined. Phase III trials in this setting had not demonstrated a survival advantage in CCA, but these studies have included a range of tumor types (including pancreatobiliary, gallbladder and ampullary carcinomas) and have failed to show sufficient power to identify a survival difference specifically in CCA [41, 42]. However, recently the results of the phase III UK BILCAP trial have been presented [43, 44]. This large phase III randomized trial recruited patients with resected biliary cancer including 368 (plus 79 gallbladder carcinomas) cholangiocarcinoma patients and randomized between no adjuvant chemotherapy or 6 mo application of oral drug capecitabine. Patients treated with capecitabine showed an improved overall survival [53 mo vs 36 mo HR = 0.75 (95% CI: 0.58–0.97; *P* = 0.028)]. The results will lead to adjuvant chemotherapy with capecitabine being adopted as a potential therapeutic option in resected biliary cancers, but the overall study is nevertheless negative according to the intention- to- treat- population. We have also no data about the radicalness of surgery especially in gallbladder carcinoma patients within the British study. Only macroscopically complete resection with curative intent was needed in BILCAP.

To conclude, there are trends for an improvement in OS due to adjuvant chemotherapy, but data demonstrating a significant improvement for adding adjuvant therapy after a curative resection are lacking [44, 45]. Liver transplantation is not a standard treatment for CCA due historically high relapse rates and donor shortage. More modern series have reported more encouraging results [46]. Potential candidates, such as patients with poor hepatic reserve for extended hepatectomy or those with a localized but irresectable perihilar biliary tract carcinoma should be enrolled on to suitable clinical studies. Locoregional therapies, including radiotherapy, photodynamic therapy, chemo/radio-embolization and radiofrequency ablation may have a role in locally advanced malignancies or patients who are surgically not fit. There is a lack of comparative clinical study evidence to support any of these options improving survival compared to standard of care systemic therapy [8]. However, retrospective and phase II data suggest a promising rate of local control by adding radiotherapy in the management of ICC, and warrants investigation in the future [47, 48].

Because of high rates of disease recurrence and poor survival rates in IGBC and ICC/ECC following surgical resection and the inadequacy of treatment modalities in the pure adjuvant therapy there is a need for an earlier intervention in the course of the disease. Due to the prognostic improvements of patients in other tumor entities (gastric, colorectal e.g. [49, 50]) treated with neoadjuvant or perioperative therapy there is a strong rationale to use these concepts in biliary and gallbladder cancers.

The current proposed phase III GAIN study investigates whether induction chemotherapy followed by radical resection in ICC/ECC and re-resection in IGBC (and – if possible – postoperative chemotherapy) prolongs overall survival compared to radical surgery alone for incidental gallbladder carcinoma and primary resectable or borderline resectable cholangiocarcinoma. Utilizing a neoadjuvant approach including a second radical surgery will help to raise awareness for the necessity of radical surgery, especially second radical completion surgery in IGBC and improve the adherence to the guidelines.

## Data Availability

Not applicable.

## Abbreviations

ACO: Assoziation chirurgische Onkologie
AIO: Arbeitsgemeinschaft Internistische Onkologie- Working group of Medical Oncologists
CALGP: Chirurgische Arbeitsgemeinschaft Leber- Galle- Pankreas
Cis: Cisplatin
DFG: Deutsche Forschungsgemeinschaft (German Research Foundation)
EORTC: European Organization for Research and Treatment of Cancer
EORTC-QLQ-C30: European Organization for Research and Treatment of Cancer Quality of Life Questionnaire-C30
Gem: Gemcitabine
QoL: Quality of Life
